# Evaluating Senegal's COVID-19 surveillance system for early detection and response: lessons from the Keur Massar district, March 03, 2020 to May 31, 2022

**DOI:** 10.1186/s12889-024-20692-6

**Published:** 2024-11-22

**Authors:** Amady Ba, Jerlie Loko Roka, Mbouna Ndiaye, Mamadou Sarifou Ba, Boly Diop, Omer Pasi

**Affiliations:** 1 Centers for Disease Control and Prevention, Georgia, United States; 2 Ministry of Health, Dakar, Senegal; 3 African Field Epidemiology Network (AFENET), Kampala, Uganda

**Keywords:** Evaluation, Surveillance system, COVID-19, Senegal

## Abstract

**Background:**

The COVID-19 pandemic highlights the importance of strong surveillance systems in detecting and responding to public health threats. We sought to evaluate attributes of Keur Massar district's existing COVID-19 surveillance system.

**Method:**

A descriptive, cross-sectional study was conducted in June 2022; desk review covered data collected from March 03, 2020 to May 31, 2022 in 18 health posts. Data were collected using a standardized questionnaire completed during a face-to-face interview and a desk review of surveillance data gathered from different notification platforms (Excel, ODK, DHIS2 aggregated, and tracker). Study was conducted in Keur Massar department, in the Dakar region. We conducted face-to-face interviews with 18 nurses in June 2022. We utilized a standardized, semi-structured questionnaire adapted from CDC guidelines for surveillance evaluation.

**Results:**

All 18 head nurses targeted, responded to the questionnaire, with an average age of 41.5 years and 63% aged between 30 and 44. The sex ratio (M/F) was 0.6, and respondents had an average of 15.1 years of experience. All nurses were involved in COVID-19 surveillance and had notified at least one suspected case.

While 39% conducted COVID-19 data analysis, 55.6% received feedback from the national level. The usefulness score for the surveillance system was 77.7, with the lowest score (72.9) related to describing the pandemic’s magnitude. Simplicity scored 63.3, with low scores for the availability of guidelines (0) but high scores for training and equipment (94.4). Acceptability scored 76.6, with strong support for COVID-19 surveillance but weak community involvement (48.6).

While no cases were reported through the DHIS2 aggregated platform, 1327 PCR-positive SARS-CoV-2 cases were reported through the national Excel sheet and 278 PCR-positive cases were reported through the COVID-19 DHIS2 tracker during the same period. Timeliness varied, averaging 3 days using ODK and 7 days with the national Excel sheet, with a combined average of 5 days across both systems.

**Conclusion:**

The study highlights challenges in COVID-19 surveillance due to limited human resources, multiple data systems, and delays in notification. While most nurses were trained and equipped, gaps in data quality, timeliness, and community support emphasize the need for streamlined processes and increased workforce capacity.

**Supplementary Information:**

The online version contains supplementary material available at 10.1186/s12889-024-20692-6.

## Background

The SARS-CoV-2 pandemic spread worldwide shortly after it started in late 2019. By 28 February 2022, there had been over 430 million laboratory-confirmed cases. During this time, Africa registered nearly 8 million confirmed cases with a 2% fatality rate [1]. To objectively compare statistics obtained from different disease surveillance systems, epidemiologists and public health professionals should consider using consistent or standardized parameters such as case definition, screening approaches, testing strategies, and contact tracing. The World Health Organization (WHO) defined epidemiological surveillance as “the ongoing and systematic collection, analysis, and interpretation of health data in the process of describing and monitoring a health event” [2]. The main objective of disease surveillance is to improve early detection capacity and effective response to diseases and other public health threats; therefore, a strong surveillance system becomes critical during any outbreak [3]. The ideal surveillance system provides real-time data not only for early detection, but also for timely notification to inform decision making. The COVID-19 pandemic revealed some weaknesses in the surveillance system in African countries that may have impacted decision making for the outbreak response management [4].

In 44 out of the 54 African countries, surveillance was conducted using the Integrated Disease Surveillance and Response (IDSR) strategy in 2019 [5]. The integration consists of harmonizing different methods, software, and case definitions in order to have comprehensive information and to optimize the efforts of different disease programs to prevent and manage public health threats [6]. During the COVID-19 pandemic, multiple types of COVID-19 surveillance including case-based routine surveillance, active surveillance, and syndromic surveillance were applied in different worldwide regions [7]. Countries used different types of surveillance depending on policies and available resources to reach objectives. Early warning active surveillance, a combination of community-based surveillance, cluster investigations, environmental surveillance, pharmaceutical vigilance, and notifiable disease screening and reporting at various sites and contexts, was recommended by the WHO and demonstrated to be an effective tool in controlling COVID-19 outbreaks [8–10].

Periodical evaluation of an established surveillance system is critical to ensure that the system aligns with its intended objectives and is operating efficiently [11]. In addition, surveillance evaluations provide information on required adjustments to improve the system [12]. COVID-19 surveillance evaluation using standardized guidelines was assessed in very few African countries with different levels of performance regarding attributes that were evaluated [13–15]. This highlighted the gaps in COVID-19 surveillance identified by the evaluation. Closing these gaps is a crucial step to efficiently improving surveillance systems.

Senegal set up a surveillance system to support COVID-19 case reports and prevention in February 2020, but no formal surveillance system evaluation was conducted. In this manuscript, we aim to describe and evaluate the attributes of existing COVID-19 surveillance systems in Senegal.

## Methods

### Evaluation design and period

We conducted a descriptive cross-sectional study to gather the perspectives of nurses working in the district health post regarding key attributes of the COVID-19 surveillance system. Additionally, a desk review, which constitutes secondary research, was conducted to assess the data quality dimensions of completeness, internal consistency, external comparisons and external consistency, within the COVID-19 surveillance system used in the district of Keur Massar, in the Dakar region of Senegal.

The evaluation retrospectively assessed data collected from the first laboratory-confirmed case of COVID-19 in Senegal on March 3, 2020, to the end of May 2022.

### Study site and population

The Department of Keur Massar consists of the health districts of Yeumbeul and Keur Massar.

It covers an area of 51 km^2^ with a density of 34,188 inhabitants per km^2^ in Yeumbeul and 8,262 inhabitants per km^2^ in Keur Massar for an estimated total population of 627.925 inhabitants in 2022, with 20 health facilities including two health centers and 18 health posts.

The Senegalese health system is organized according to a three-level pyramid structure: the national level, which is responsible for giving directives and guidance to all other levels; the intermediate level, which corresponds to the health regions and plays a coordination role between the national and the lowest level; and, the operational level, which corresponds to the districts, the lowest level of the health hierarchy. The operational level includes the health district team responsible for managing the district, as well as all the private and public health structures that provide direct patient care.

### COVID-19 surveillance system in Senegal

Senegal established a surveillance system during the COVID-19 pandemic using both passive and active surveillance, with the goal of providing information for decision-making. The system used standard case definitions adapted from the WHO case definitions and evolved its detection strategy over time. At the outset, all symptomatic patients and contacts of individuals diagnosed with SARS-CoV-2 infection underwent systematic testing. However, beginning from end of June 2020, testing was limited to symptomatic individuals and vulnerable contacts, specifically those with comorbidities and those aged 60 years and above. Initially, only real-time Polymerase Chain Reaction (rtPCR) tests were used. Starting in July 2021, antigenic rapid diagnosis tests (Ag-RDT) were introduced in addition to rtPCR. The response data collection was carried out using four tools, the national COVID Excel sheets, the Open Data Kit (ODK), the District Health Information System 2 (DHIS2) tracker and aggregat; the implementation of each tool started at different stages of the outbreak (see Fig. 2). Data encoding for the Excel sheet was carried out at the national level by the Incident Management System (IMS) team, while at the operational level, the surveillance focal point was responsible for data encoding on all other platforms. The national COVID Excel sheet contains the nationwide positive cases tested by rtPCR in the national reference laboratories. It collected data on each case's socio-demographic profile, exposure (including travel history), symptoms, underlying conditions, rtPCR test and results, hospitalization, and the patient's final outcome. It served as a temporary database with the intention of being replaced by the DHIS2 tracker, the platform chosen as the official system by the Ministry of Health and supported by partners. The ODK tool, an open-source app for data collection, was implemented to capture information related to patients tested using the Ag-RDT. It was used to collect information on each patient's socio-demographic profile, symptoms, COVID-19 vaccination status, Ag-RDT test, and results. The DHIS2 tracker, which is an application within the DHIS2 platform for the collection of individual-level (or case-based) linear data, was implemented to capture information on both suspected cases, rtPCR-confirmed and their contacts The tracker collected information on patient socio-demographic profile, symptoms, underlying conditions, case type, treatment, confirmed case contact history, eligibility for home-based management, vaccination status, quarantine, hospitalization, caregiver information, sample type, and patient outcome. The DHIS2 aggregated system for COVID-19 surveillance, compiled data on the weekly numbers of suspected cases, tests conducted, rtPCR confirmed cases, and deaths.

Data compilation and analysis for all three tools were primarily conducted at the national level, involving the IMS team and the routine surveillance department team. The resulting reports were shared with all relevant stakeholders at the regional and operational levels.

### Case definitions

The case definition for COVID-19 evolved over time, with the first issued in April 2020 and the latest in July 2021. As such, the detected cases reflect the case definition in place during July 2021. Below, we presented the case definition available from July 2021.

A *suspected case* was a) a person who meets both the clinical and epidemiological criteria *Clinical criteria* included acute onset of fever and cough; or, acute onset of at least three of the following signs or symptoms: fever, cough, general weakness/fatigue, headache, myalgia, sore throat, runny nose, dyspnea, anorexia /nausea/vomiting, diarrhea, altered mental state. *Epidemiological criteria* included residing or working in a high-risk virus transmission area (closed residential settings, healthcare settings, including healthcare facilities or within the community) at any time within the 14 days preceding the onset of symptoms; or, residing or traveling in a community transmission area at any time within the 14 days preceding the onset of symptoms.

b) A patient with severe acute respiratory illness (SARI): severe acute respiratory infection with a history of fever or a measured fever of > = 39 °C; and cough; with onset in the last 10 days; and requiring hospitalization.

*Probable case* was defined as one of the following: a) A patient who meets the clinical criteria (but not epidemiologic criteria) mentioned above and who is a contact of a probable or confirmed case or linked to a COVID-19 cluster; b) A suspected case with chest imaging results suggestive of COVID-19; c) A person presenting with recent onset anosmia (loss of smell) or ageusia (loss of taste), in the absence of any other identified cause; or, d) Unexplained death in an adult with preceding respiratory distress, who is a probable or confirmed contact or linked to a COVID-19 cluster.

*Confirmed case* was defined as one of the following: a) A person with a positive rtPCR; or, b) A person whose SARS-CoV-2 Ag-RDT is positive and who meets the probable case definition; or who meets the criteria for suspicion in a or b.

c) An asymptomatic person with a positive SARS-CoV-2 Ag-RDT who is a contact of a probable or confirmed case.

### Data collection

#### Desk review

We reviewed existing health district documents to assess the quality of the COVID-19 surveillance data from March 3, 2020 (when Senegal reported the first laboratory confirmed COVID-19 case) to May 31, 2022 (when Senegal was reporting few to no COVID-19 cases per week). The data sources included the ODK tool (COVID-19 Ag-RDT dataset), the national COVID-19 Excel sheet, and both the DHIS2 tracker and aggregated data for COVID-19.

#### Surveys

In June 2022, we conducted face-to-face interviews with one head nurse from each of the 18 health posts in the Keur Massar department. We utilized standardized, semi-structured questionnaires adapted from CDC guidelines [16]. The questionnaires aimed to assess nurses’ perceptions of the surveillance system's usefulness and select attributes, such as simplicity, acceptability, data quality, and timeliness. The study also evaluated COVID-19 surveillance functions (detection, confirmation, analysis, feedback, availability of standard guidelines, and staff training) based on the WHO guideline for surveillance evaluation [2]. To evaluate system attributes, participants responded to questions using either a binary response (yes/no) or a Likert point scale. Demographic data for the participants was also collected. All responses were recorded using Open Data Kit software (ODK). Participants provided consent and received no incentives for participation.

#### Definitions for Attributes used in Surveillance Evaluation

We used the US Centers for Disease Control and Prevention (CDC) surveillance system evaluation guide standard definitions for usefulness, simplicity, acceptability, data quality and timeliness [16], as mentioned in Table 1.

**Table 1:** Usefulness and selected attributes definition, evaluation measures, and methods

Usefulness and attributes	Definition	Evaluation measures	Method of evaluation	Documents
**Usefulness**	Contributes to the prevention and control of adverse health-related events	-Contribute to the prevention and control of adverse occupational health events and to an improved understanding of the public health implications of such events	Survey	
	Helps to determine that an adverse health-related event previously thought to be unimportant is actually important	-Usefulness of the surveillance system perceived by stakeholders	Survey	
**Simplicity**	The simplicity of structure and ease of operation to reach its objectives	- A well-defined drawn case management flowchart -Simplicity of the case definitions, it’s understanding, and applicability for the health workers -Few organizations requiring data reports	Desk review Survey	
**Acceptability**	Willingness of persons and organizations to participate in the surveillance system	-Willingness of stakeholders to collaborate with the program -Stakeholders' participation in program activities	Survey Survey	Database
**Data quality (for key variables)**	Completeness and validity of the data recorded; Consistency	Data completeness will be evaluated by calculating the percentage of “unknown” or “blank” responses in each dataset. We considered low quality missing data and inconsistency above 5%	Desk review	Database from national Excel sheet, ODK, DHIS2 tracker
Timeliness	Speed between steps in the surveillance system	Detection timeliness was the difference in days between the onset date and the notification date Detection within 7 days of onset date was considered to be timely and a good performance	Desk review	Database from national Excel sheet, ODK, DHIS2 tracker

### Data management and analysis

#### Surveys

Responders’ years of experience and age averages with standard deviations were calculated. The Likert scale scores from the survey were coded from 0 to 5 (0 = Unknown/NA, 1 = Totally disagree, 2 = Disagree, 3 = Neither agree nor disagree, 4 = Agree, 5 = Very much agree), with Boolean questions coded as 1 and 0 for Yes/No responses.

We used the following formula to calculate a score for each indicator that falls between 0 and 100. Scores were calculated among those who had a response other than “Unknown/NA/NSP”:

$${\varvec{I}}{\varvec{n}}{\varvec{d}}{\varvec{i}}{\varvec{c}}{\varvec{a}}{\varvec{t}}{\varvec{o}}{\varvec{r}}\boldsymbol{ }{\varvec{a}}{\varvec{c}}{\varvec{t}}{\varvec{u}}{\varvec{a}}{\varvec{l}}\boldsymbol{ }{\varvec{S}}{\varvec{c}}{\varvec{o}}{\varvec{r}}{\varvec{e}}=\boldsymbol{ }\sum \left({\varvec{F}}{\varvec{r}}{\varvec{e}}{\varvec{q}}{\varvec{u}}{\varvec{e}}{\varvec{n}}{\varvec{c}}{\varvec{y}}\boldsymbol{ }\boldsymbol{*}{\varvec{s}}{\varvec{c}}{\varvec{a}}{\varvec{l}}{\varvec{e}}\boldsymbol{ }{\varvec{v}}{\varvec{a}}{\varvec{l}}{\varvec{u}}{\varvec{e}}\right)/\boldsymbol{ }\left(\mathbf{n}\boldsymbol{*}\mathbf{M}\mathbf{a}\mathbf{x}\boldsymbol{ }\left(\mathbf{s}\mathbf{c}\mathbf{a}\mathbf{l}\mathbf{e}\mathbf{v}\mathbf{a}\mathbf{l}\mathbf{u}\mathbf{e}\right)\right)$$

The overall attribute value was determined by computing an equally weighted average of the indicator scores within each component.

For the final classification we considered the following thresholds: score less than 60 = weak performances; between 60 and 80 = middle performances; more than 80 = good performances.

Statistical analyses were performed using R and Excel.

#### Desk Review

 For each case key variable (date of onset, sex, age of cases and at least one symptom specified in the case definition) the percentage of missing values were calculated to assess data completeness, with a score of 1 assigned when missing values were equal to or less than 5%, and a score of 0 when they exceeded 5%. In addition, consistency was assessed for the subset of four key variables (date of onset, sex, age of cases and at least one symptom specified in the case definition) in each of the previously listed datasets from March 3, 2020, to May 31, 2022. Timeliness was assessed by calculating the difference in days between illness onset date and notification post laboratory results date in each electronical platform, which made the data accessible at all health pyramid levels.

## Results

All 18 head nurses responded to the questionnaire. The average age was 41.5 years, with a majority (63%) between 30 and 44 years of age. Sex ratio (M/F) was 0.6, and respondents, with an average of 15.1 years of experience as nurses, and all reported being involved in COVID-19 surveillance activities. All respondents had notified at least one suspected case of COVID-19 to their respective hierarchical level.

### Surveillance functions capacities of survey respondents

**Fig. 1:**
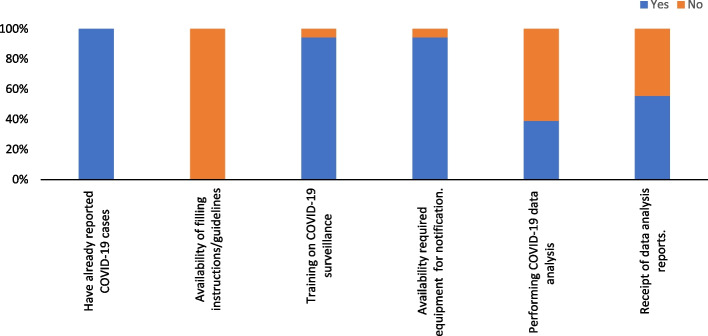
Training and equipment for core surveillance functions by 18 survey responders. Red arrows indicate the starting date of the implementation of each database

Ninety-four percent (17/18) of respondents declared they were trained on COVID-19 surveillance, and the same percentage declared they had the necessary equipment to appropriately conduct COVID-19 surveillance. Additionally, 39% (7/18) of them conducted COVID-19 data analysis, while 55.6% received feedback from the national level. (Fig. 1).

All cases were collected at the operational level, and the equipment (e.g., computer, wifi) availability score for notification was 94. Case definitions and case report forms were shared by the national level with all operational level staff, and the scores for the understanding and application of the case definitions were 74.5 and 76.5 respectively (Table 2).

**Table 2:** Usefulness, and selected attributes and indicators score

	**Frequency**^**a**^	**Actual score**^**b**^	**Max score**	**Averages**
**Usefulness**		474	610	**77.7**
Description Covid-19 magnitude	17	62	85	72.9
Description of sociodemographic characteristics and vaccination status	15	58	75	77.3
Implementation of public health response	18	72	90	80.0
Data collected is important for COVID-19 surveillance at KM	18	72	90	80.0
Sufficient information for public health decision making	18	69	90	76.7
Data used for public health decision making	18	69	90	76.7
Use to measure the impact of prevention measures	18	72	90	80.0
**Simplicity**		**371**	**535**	**63.3**
Availability of filling instructions and guidelines	18	0	18	0
Training/guidance for the detection of COVID-19	18	17	18	94.4
Availability of the necessary equipment for the notification	18	17	18	94.4
Analysis of COVID-19 data performed	18	7	18	38.9
Receipt of data analysis reports	18	10	18	55.6
Ease of filling out the questionnaire	12	48	60	80.0
Simplicity of filling instructions and guidelines	3	12	15	80.0
Easy to understand training/guidance on COVID-19 monitoring	18	65	90	72.2
Easy to understand platform operation	11	41	55	74.5
Easy to understand case definition	17	65	85	76.5
Easy to apply case definition in practice	11	41	55	74.5
Easy to perform COVID-19 data analysis	17	48	85	56.5
**Acceptability**		**383**	**513**	**76.6**
Contribution to COVID-19 monitoring valued	18	18	18	100
Satisfaction with level of involvement in COVID-19 monitoring	13	46	65	70.8
Colleague interest in COVID-19 monitoring activities	18	72	90	80.0
Covid19 is a public health issue	18	72	90	80.0
Different platforms useful for managing detected COVID-19 cases	18	72	90	80.0
Community support for COVID-19 surveillance	14	34	70	48.6
Contribution to COVID-19 monitoring valued	18	69	90	76.7
**Data quality**		**136**	**188**	**64.6**
Adequacy of the training/orientation provided for Covid19 monitoring	18	72	90	80.0
Adequacy of allocated time for COVID-19 monitoring data management	18	59	90	66.0

^a^Number of responders

^b^actual Score = Σ (Frequency*scale value)/ (n*Max (scale value))

### Attributes analysis

#### Usefulness

Overall score for usefulness was 77.7 with the highest scores on indicators related to public health measures, implementation, and the established surveillance system used in measuring the impact of prevention measures (Table 2). Furthermore, all indicators related to this attribute scored above 60, with the lowest recorded score of 72.9 being for usefulness of the system to describe the magnitude of the COVID-19 pandemic (Table 2).

#### Simplicity

Overall assessment score of COVID-19 surveillance system simplicity was 63.3. The lowest scores were obtained for the availability of guidelines and instructions (0). Additionally, the reported ease of COVID-19 data analysis was 56.5 and the highest scores were registered for both training for COVID-19 detection and equipment availability for notification, both scoring 94.4 (Table 2).

#### Acceptability

All survey respondents agreed with the fact that COVID-19 is a public health issue in their district and with their level of involvement in the COVID-19 surveillance system. The overall score is 76.6, with the highest average obtained for the respondents' statement on COVID-19 monitoring (100). However, community support for COVID-19 surveillance is low, scoring 48.6 (Table 2).

#### COVID case data quality

The Excel sheet, DHIS2 tracker, ODK, and DHIS2 aggregated data included 43, 69, 26, and 4 variables, respectively. Of these, 26 variables related to patient socio-demographic information, symptoms, date of illness onset, hospital admission, sample dates and types, laboratory results, patient comorbidities, and the patient's status were common between the national COVID-19 Excel sheet and the DHIS2 tracker. However, the Excel sheet contained details on exposures that were not recorded in the tracker, while the tracker held information related to clinical management, healthcare providers, and vaccination status that was not found in any other source. Additionally, the ODK included socio-demographic data, vaccination status, the type of administered vaccines, and COVID-19 antigen test information. All the variables in the DHIS2 aggregated database? could have originated from either the Excel sheet or the tracker.

The overall score for data quality was 64.4 pooling all indicators we assessed for the attribute. Less than 5% of the data for date of onset in ODK as well as sex in both ODK and Excel were missing. Age and symptoms also were missing less than 5% of the time in both the Excel and ODK data management platforms (Table 2).

During the study period, no cases were reported through the DHIS2 aggregated system for the Keur Massar district, as that platform has never been used in the district. For that same period, a total of 1,412 SARS-CoV-2 positive cases were reported through the ODK tracker and 1,327 through the Excel sheet, as shown in Table 3; the differences between the number of reported cases by the two platforms varied by calendar year. Of the 278 cases reported by the COVID-19 DHIS2 tracker, 100% (278/278) of the values for the key explored variables (sex, date of onset, and symptoms) were found to be missing. The percentage of missing values for these key variables through other platforms (Excel and ODK) is reported in Table 3. None of the key variables were completed at 100%, and only one variable (sex) had at least 95% completeness across all platforms. Less than 5% of the illness onset date and symptom variables in the ODK platform and the age variable in the Excel were missing. (Table 3).

**Table 3:** Evaluation of COVID-19 surveillance data completeness and availability, Keur Massar department, 2020–2022

	**2020**^**a**^		**2021**^**a**^		**2022**^**a**^	
Required variables	**ODK**	**Excel** **n (%)**	**ODK**	**Excel**	**ODK**	**Excel**
Total number of cases reported in data management platform	0	321	1028	934	384	72
Symptoms	-	275 (85.7)	1006 (97.9)	605 (64.8)	384 (100)	41 (56.9)
Onset date	-	179 (55.8)	981 (95.4)	57 (6.1)	384 (100)	0
Sex	-	316 (98.4)	1028 (100)	930 (99.6)	384 (100)	72 (100)
Age	-	309 (96.3)	281 (27.3)	908 (97.2)	366 (95.3)	72 (100)

^a^All key variables were missing from the COVID-19 DHIS2 tracker

n: number of reported cases

%: proportion of available data

## Timeliness

**Fig. 2:**
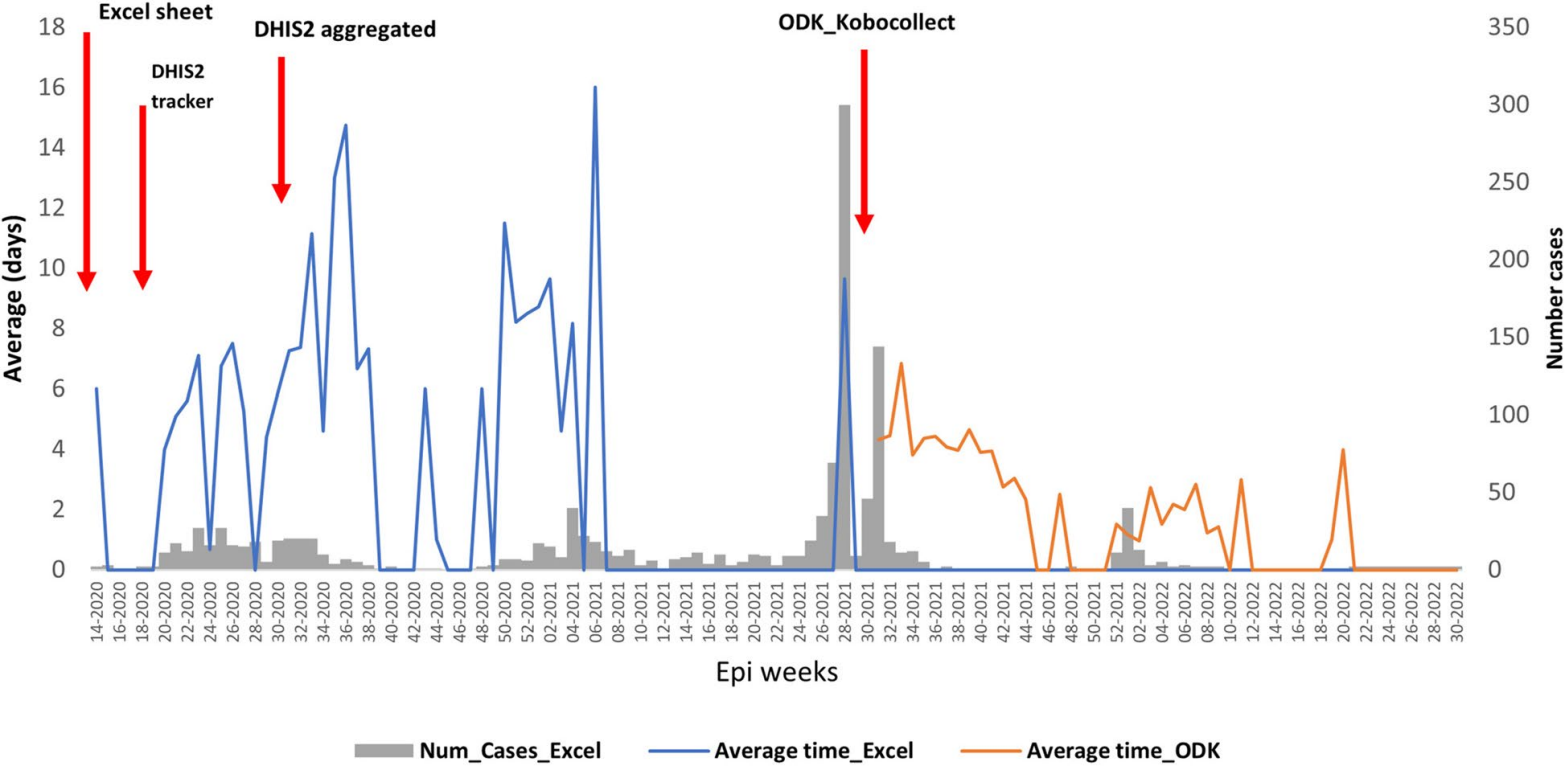
Average time between onset date of symptomatic COVID-19 and case reporting, Keur Massar department, Dakar, Senegal, March 03, 2020 to May 31, 2022

Timeliness between illness onset date and notification varied depending on the platform. Indeed, on average it took 3 days using the ODK platform, while it went up to 7 days using the national Excel sheet starting from symptoms onset to detect and notify COVID-19 cases in the study area. Overall daily average duration in both notification platforms combined was 5 days. Detection timeliness varied over time, but longer duration did not appear to coincide with higher number of confirmed cases (Fig. 2).

## Discussion

This study examines the COVID-19 surveillance system in place in the Keur Massar department of Senegal, and evaluates its performance in terms of usefulness, simplicity, acceptability, data quality, and timely detection of cases through review of reported case data and interviews of the head nurse for each of 18 health districts.

The analysis of surveillance core functions revealed that the majority (94%, 17 out of 18) of responders in the study setting were trained and equipped to perform COVID-19 surveillance. National-level online training sessions were conducted, targeting operational staff whenever important new directives were issued. These training sessions covered various areas, including case definitions, COVID-19 Ag-RDT usage, and data management platform utilization. A higher percentage of responders in our study site were trained compared to many other African countries in their COVID-19 surveillance efforts [17, 18]. The high training rate may be attributed to the surge in capacity brought by both the Senegal government and partners about COVID-19, which demanded swift and comprehensive actions. Despite the majority of responders in our study declaring that the case definition was simple and easy to apply, we did not specifically explore their knowledge of the COVID-19 case definitions, which could potentially impact frequency of case detection [19]. Our analysis revealed a lack of available guidance manuals for users to reference when performing tasks. Such manuals could help bridge the knowledge gaps arising from less attentive participation, especially in the context of online training during the COVID-19 pandemic. This situation may be attributed to the emergency nature of the pandemic and the urgency with which activities had to be conducted within a very short time period. However, there is a need to explore the extent to which surveillance officers effectively used manuals when available and how this contributed to improving their daily work.

While routine surveillance through the Integrated Diseases Surveillance and Response (IDSR) implementation follows a well-structured flow of data collection and transmission in the country, the structure differed during the COVID-19 emergency, and our analysis identified multiple databases for case notification. Among these databases, only ODK was fully complementary, capturing information related to Ag-RDT that was not recorded elsewhere. The DHIS2 tracker proved to be cumbersome for daily management, requiring the completion of three different forms for each confirmed case. Furthermore, the WHO recommended its use only for the initial cases, and the country's surveillance system lacked the flexibility to accommodate this recommendation. Ultimately, the DHIS2 aggregated value would be more apparent if it could either compile information directly from existing data within the system or fully replace the DHIS2 tracker.

This variation likely contributed to confusion at the operational level. Overall, while some of these platforms were complementary, such as the COVID-19 DHIS2 tracker and ODK, others collected mostly the same type of data, which increased health personal including workload and potentially affected data quality, specifically consistency and missing values. The multiplicity of datasets, combined with the increased workload on staff without a corresponding increase in human resources, are key factors that may have directly affected data quality on the ground. An analysis conducted in four African countries, including Senegal, highlighted the shortage of human resources as a common challenge in managing the COVID-19 pandemic across all these countries. [20]. Therefore, harmonizing the data collection system and ensuring appropriate human resource surge capacity adapted to the emergency and at an early stage would not only be beneficial but also timesaving and much more efficient. Similar discrepancies were identified from the analysis performed on COVID-19 surveillance data in Portugal, even though they were found in the same database but issued at different periods of time, making the existence of multiple databases even riskier [21]. Previous analyses have shown that poor data quality can bias evidence-based decision-making, underscoring the necessity for implementing best practices to improve data quality [22].. Finally, we found that only a few responders (39%) were engaged in COVID-19 data analysis. This may indicate a lack of ownership at the operational level, where individuals do not instinctively utilize the data, they generate to make decisions, even though they have a better understanding of the local situation. Health authorities at various levels should make efforts to promote and encourage the involvement of operational staff in data analysis for decision-making. Implementing a bottom-up approach to decision-making would be more appropriate and beneficial for the entire system. This way, decisions can be made based on insights gathered from those working closely with the local context, resulting in more informed and effective actions. This is particularly important in our context, where background information may not be adequately collected, and decision-making driven solely by data using algorithms may not be the most relevant, as suggested elsewhere [23].

The objective of the COVID-19 surveillance system is to detect cases at an early stage and report them in a timely way in order to mitigate disease spread in the community [10]. However, we found that the surveillance system performed timely detection of cases only moderately well and was weak when considering the timeliness of reporting using only the national COVID-19 Excel sheet. This can be because only a few people were encoding data for the entire country, which became quickly overloaded when the number of cases reached a hundred a day. We suggest two solutions to this problem: having enough human resources at the national level to encode data on time (in case the same design of data management is established for future outbreaks) or establish a functional data entry at the local level with the appropriate resources. The COVID-19 surveillance system in the Keur Massar department detected 1,327 patients while the nationwide seroprevalence survey found a 44% (1,687,405 estimated number of COVID-19 infections) infection rate in the Dakar region, suggesting significant under-detection by the surveillance system [24]. The high documented proportion of asymptomatic COVID-19 cases can partially explain this result as the surveillance system was mostly passive, it could only detect symptomatic patients who self-reported or visited health facilities [25].

## Limitations

This evaluation was conducted in one district and may not represent the COVID-19 surveillance system in other areas. Caution should be taken when applying the results beyond the specific district where it was conducted.

Self-reported data may be biased or erroneous due to the respondents' perceptions, experiences, or feelings. The survey nurses were experienced and trained, but the respondents' answers may still reflect their perceptions rather than objective reality. Therefore, the survey results should be interpreted with caution and considered alongside other data sources for a comprehensive understanding of the situation.

We primarily conducted quantitative analysis, and supplementing it with qualitative data collection methods, such as focus groups, could have provided us with deeper insights for better understanding the system and interpreting our results.

The lack of a unique patient identifier makes it impossible to track duplications in various linear data collection tools and properly identify the platform that captures the maximum number of unique patients.

## Conclusion

In conclusion, this manuscript examined the performance of the COVID-19 surveillance system in the Keur Massar department, Senegal. The majority of responders reported being well-trained and equipped, but their depth of knowledge in case definition needs further exploration. The lack of available guidance manuals during the pandemic might have affected data quality. Harmonizing multiple databases and promoting operational staff involvement in data analysis are crucial for efficient decision-making. Timely detection can be improved by allocating sufficient resources at the national level or adopting a local data entry strategy. The surveillance system exhibited under-detection, possibly due to its passive nature. To enhance effectiveness, active case detection and a decentralized approach could be considered. Implementing these measures will fortify the system's capabilities in mitigating disease spread by improving the detection process and decision-making during future outbreaks.

## Implications for policy & practice

According to our analysis, the COVID-19 surveillance system needs improvement to ensure its effectiveness.

Training and Equipment: The high percentage of trained and equipped responders (94%) is promising and highlights the importance of continued investment in training initiatives to enhance preparedness for future outbreaks.

Guidance Manuals: The lack of available guidance manuals during the pandemic indicates a need for improved preparedness measures, including the development and dissemination of comprehensive guidance materials for responders to ensure consistent and accurate data collection.

Harmonization of Databases: The presence of multiple databases for case notification, with inconsistencies in reported positive cases, calls for urgent harmonization and integration of data sources to improve data quality and decision-making accuracy.

Data collection and management: A surge in resources, including human resources, is essential to effectively manage outbreaks and ensure staff can maintain high-quality services at all levels, particularly in surveillance. Additionally, it is important for health authorities to support data management in the field by providing harmonized and simplified tools at an early stage.

Promoting Data Analysis and Feedback: The low engagement of responders in COVID-19 data analysis underscores the importance of fostering a culture of data-driven decision-making. Authorities should encourage and support operational staff involvement in data analysis to improve surveillance effectiveness.

## Supplementary Information

Supplementary Material 1.

## Data Availability

The datasets used in the current study are available from the corresponding author and can be accessed in the provided repository.
