# Supplementary material for: Evaluating Senegal's COVID-19 surveillance system for early detection and response: lessons from the Keur Massar district, March 03, 2020 to May 31, 2022
Source: BMC Public Health. 2024 Nov 22;24:3246. doi: 10.1186/s12889-024-20692-6 (PMC11583634; doi:10.1186/s12889-024-20692-6)
Supplement: Supplementary file 1 — Supplementary Material 1. [file 12889_2024_20692_MOESM1_ESM.pdf]

Supplemental Digital Content (SDC): Questionnaire for the evaluation of COVID-19 surveillance in the Department of Keur Massar, health facility level

**GENERALE INFORMATION**		
District name	………………………………………………	
Health facility name	…………………………………………………………………	
Socio-demographics characteristics	Age (Years)	
	Sex	Male ☐ Female ☐
What's your position	□ Physician/Provider<br>□ Laboratory technician<br>□ Surveillance focal point (EPI/SE FP)<br>□ Other, specify:	
What is your seniority in the position?		
What is your education level?	□ Master<br>□ State diploma<br>□ Certificate<br>□ Other, please specify:................................	

Choose the option that best matches your opinion and put a comment at the end of the section	
Q1. Have you ever reported any cases of COVID-19?	□ Yes<br>□No
Q3. What data sources do you use to complete the questionnaires available in each platform?	
Q4. On average, how much time do you spend completing the COVID-19 questionnaire?	
**SIMPLICITY**	
**Questions**	
Q1. Are instructions and guidelines for completing the questionnaire for each platform available?	□ Yes<br>□No
Q2. Have you been trained for COVID-19 surveillance (Platforms and case definition)?	□ Yes<br>□No
Q3. Do you have all the equipment (computer, phone or tablet; internet/Wi-Fi connection) needed to transmit data across each platform?	□ Yes<br>□No
Q4. Have you ever analyzed the COVID-19 data collected across each platform?	□ Yes<br>□ No

Q4.1 If analysis of COVID-19 data is done, on average, how much time do you spend analyzing the COVID-19 data collected across each platform	
Q4.2 if COVID-19 data analysis is done, what methods do you use to analyze the data from each platform?	
Q5. Did you receive the report of the analysis of the data from each platform done by the central level?	□ Yes<br>□ No
Q6. The variables to be filled in on each platform are easy to complete	□ Totally disagree<br>□Disagree<br>□Neither agree nor disagree<br>□Agree<br>□Very much agree<br>□NA<br>□Unknown
Q7. Are the instructions and guidelines for completing the questionnaire for each platform easy to understand?	□ Totally disagree<br>□Disagree<br>□Neither agree nor disagree<br>□Agree<br>□Very much agree<br>□NA<br>□Unknown
Q8. Do you find your COVID-19 surveillance training/orientation (Platforms and case definitions) easy to understand?	□ Totally disagree<br>□Disagree<br>□Neither agree nor disagree<br>□Agree<br>□Very much agree<br>□NA<br>□Unknown
Q9. How easy was it for you to understand how each platform works to collect and send COVID-19 data?	□ Totally disagree<br>□Disagree<br>□Neither agree nor disagree<br>□Agree<br>□Very much agree<br>□NA<br>□Unknown
Q10. Were the COVID-19 case definitions (suspect and confirmed) including the use of RDTs easy for you to understand?	□ Totally disagree<br>□Disagree<br>□Neither agree nor disagree<br>□Agree<br>□Very much agree<br>□NA<br>□Unknown
Q12: Do you consider that the information flow in the COVID-19 surveillance was simple and fast	□ Totally disagree<br>□Disagree<br>□Neither agree nor disagree

	□Agree<br>□Very much agree<br>□NA<br>□Unknown
Q13. If analysis of COVID-19 data is done, do you consider this analysis of COVID-19 data to be easy?	□ Totally disagree<br>□Disagree<br>□Neither agree nor disagree<br>□Agree<br>□Very much agree<br>□NA<br>□Unknown
Q14 :Your training/orientation was on which platform(s) were you trained?	□ Tracker Covid19<br>□ DHIS2 aggregate<br>□ ODK
Q15: Which platform(s) do you find easiest to use	□ Tracker Covid19<br>□ DHIS2 aggregate<br>□ ODK<br>□ None
**Comments**	
**ACCEPTABILITY**	
Q1. Are you involved in the monitoring of COVID-19	□ Yes<br>□ No
Q2. Do you think that your contribution and inputs to the COVID-19 monitoring system are considered important	□ Totally disagree<br>□Disagree<br>□Neither agree nor disagree<br>□Agree<br>□Very much agree<br>□NA<br>□Unknown
Q3. Are you satisfied with your level of involvement in Covid19 surveillance for case detection at your facility?	□ Totally disagree<br>□Disagree<br>□Neither agree nor disagree<br>□Agree<br>□Very much agree<br>□NA<br>□Unknown
Q4. Do fellow healthcare staff at the facility show interest in COVID-19 monitoring activities?	□ Totally disagree<br>□Disagree<br>□Neither agree nor disagree<br>□Agree<br>□Very much agree<br>□NA<br>□Unknown
Q5. Do you consider COVID-19 to be a public health problem in the district?	□ Totally disagree<br>□Disagree<br>□Neither agree nor disagree

	□Agree<br>□Very much agree<br>□NA<br>□Unknown
Q6. Do you think that the establishment of each platform was necessary for the management of detected COVID-19 cases	□ Totally disagree<br>□Disagree<br>□Neither agree nor disagree<br>□Agree<br>□Very much agree<br>□NA<br>□Unknown
Q7. Do you think that the community living in the Keur Massar district supports the implementation of Covid19 surveillance for the detection?	□ Totally disagree<br>□Disagree<br>□Neither agree nor disagree<br>□Agree<br>□Very much agree<br>□NA<br>□Unknown
**Comments :**	
**Usefulness**	
Q1. Do the data collected through each platform make it possible to describe the extent of COVID-19 in the Keur Massar/Yeumbeul district?	□ Totally disagree<br>□Disagree<br>□Neither agree nor disagree<br>□Agree<br>□Very much agree<br>□NA<br>□Unknown
Q2. Do the data collected through each platform make it possible to describe the socio-demographic characteristics and vaccination status of COVID-19 patients in the Keur Massar/Yeumbeul Health District?	□ Totally disagree<br>□Disagree<br>□Neither agree nor disagree<br>□Agree<br>□Very much agree<br>□NA<br>□Unknown
Q3. Can the data collected through each platform be used to implement a response to limit the spread of the COVID-19 epidemic in the Keur Massar/Yeumbeul district?	□ Totally disagree<br>□Disagree<br>□Neither agree nor disagree<br>□Agree<br>□Very much agree<br>□NA<br>□Unknown
Q4. Do you consider the data collected through each platform to be important for Covid19 surveillance in the Keur Massar/Yeumbeul district?	□ Totally disagree<br>□Disagree<br>□Neither agree nor disagree<br>□Agree<br>□Very much agree<br>□NA

	□Unknown
Q5. Do you consider that the data collected through each platform has produced sufficient information for public health decisions to limit the spread of COVID-19 in the Keur Massar/Yeumbeul district?	□ Totally disagree<br>□Disagree<br>□Neither agree nor disagree<br>□Agree<br>□Very much agree<br>□NA<br>□Unknown
Q6. Do you consider that the data collected through each platform was used to make public health decisions to limit the spread of COVID-19 in the Keur Massar/Yeumbeul district?	□ Totally disagree<br>□Disagree<br>□Neither agree nor disagree<br>□Agree<br>□Very much agree<br>□NA<br>□Unknown
Q7. Do you think that the data collected through each platform allows us to measure the impact of prevention actions	□ Totally disagree<br>□Disagree<br>□Neither agree nor disagree<br>□Agree<br>□Very much agree<br>□NA<br>□Unknown
Q8: Which platform(s) do you find most useful for Covid19 data collection and reporting?	□ Tracker Covid19<br>□ DHIS2 aggregate<br>□ ODK<br>□ None
Q9. Name 3 public health actions taken using the data collected through each platform	
**Comments:**	
**DATA QUALITY**	
**To be evaluated on 4 variables (Age, gender, test date, and onset date of signs);<br>Put the proportion of missing variables for each variable**	
Q1: Percentage of missing values in the Kobo	Not included in the face-to-face questionnaire
Q2: Percentage of missing values in the COVID-19 tracker	Not included in the face-to-face questionnaire
Q3: Percentage of outliers in the Kobo	Not included in the face-to-face questionnaire
Q4: Percentage of outliers in the COVID-19 tracker	Not included in the face-to-face questionnaire
Q5: Percentage of outliers in the aggregate COVID-19 DHIS2	Not included in the face-to-face questionnaire
Q6. Was the training/orientation provided for Covid19 monitoring adequate?	□ Totally disagree<br>□Disagree<br>□Neither agree nor disagree<br>□Agree<br>□Very much agree<br>□NA

	□Unknown
Q8. Do you feel that the time allocated for COVID-19 monitoring data management was adequate?	□ Totally disagree<br>□Disagree<br>□Neither agree nor disagree<br>□Agree<br>□Very much agree<br>□NA<br>□Unknown
Q9. Number of suspected cases (by case definition) of COVID-19 identified in registries during the last 3 months of the study period	Month1 :<br>Month2:<br>Month3:
**Comments :**	
**Timeliness (To be calculated)**	
Average time between the date of testing and the date of notification of the case to the higher level for each health facility during the last 3 months of the study period	Kobo Month1:<br>Kobo Month2:<br>Kobo Month3:<br>Tracker Covid19 Month1:<br>Tracker Covid19 Kobo Month2:<br>Tracker Covid19 Kobo Month3:
**Comments :**	
